# Induction of Epoxide Hydrolase, Glucuronosyl Transferase, and Sulfotransferase by Phenethyl Isothiocyanate in Male Wistar Albino Rats

**DOI:** 10.1155/2014/391528

**Published:** 2014-01-27

**Authors:** Ahmad Faizal Abdull Razis, Noramaliza Mohd Noor, Nattaya Konsue

**Affiliations:** ^1^Food Safety Research Centre (FOSREC), Faculty of Food Science and Technology, Universiti Putra Malaysia, 43400 Serdang, Selangor Darul Ehsan, Malaysia; ^2^Department of Imaging, Faculty of Medicine and Health Sciences, Universiti Putra Malaysia, 43400 Serdang, Selangor, Malaysia; ^3^School of Agro-Industry, Mae Fah Luang University, 333 Moo1 Thasud Muang, Chiang Rai 57100, Thailand

## Abstract

Phenethyl isothiocyanate (PEITC) is an isothiocyanate found in watercress as the glucosinolate (gluconasturtiin). The isothiocyanate is converted from the glucosinolate by intestinal microflora or when contacted with myrosinase during the chopping and mastication of the vegetable. PEITC manifested protection against chemically-induced cancers in various tissues. A potential mechanism of chemoprevention is by modulating the metabolism of carcinogens so as to promote deactivation. The principal objective of this study was to investigate in rats the effect of PEITC on carcinogen-metabolising enzyme systems such as sulfotransferase (SULT), N-acetyltransferase (NAT), glucuronosyl transferase (UDP), and epoxide hydrolase (EH) following exposure to low doses that simulate human dietary intake. Rats were fed for 2 weeks diets supplemented with PEITC at 0.06 *µ*mol/g (low dose, i.e., dietary intake), 0.6 *µ*mol/g (medium dose), and 6.0 *µ*mol/g (high dose), and the enzymes were monitored in rat liver. At the Low dose, no induction of the SULT, NAT, and EH was noted, whereas UDP level was elevated. At the Medium dose, only SULT level was increased, whereas at the High dose marked increase in EH level was observed. It is concluded that PEITC modulates carcinogen-metabolising enzyme systems at doses reflecting human intake thus elucidating the mechanism of its chemoprevention.

## 1. Introduction

Phenethyl isothiocyanate (PEITC) is a phytochemical with an aromatic side chain, found in cruciferous vegetables such as watercress, where it is present as a glucosinolate, so called gluconasturtiin [1]. Whenever this vegetable is interrupted, for instance during mastication, the enzyme myrosinase (**β**-thioglucoside glucohydrolase) is released and induce the conversion of gluconasturtiin into PEITC as well as in the human intestine by microbial myrosinase [2]. Epidemiological studies reported an inverse association between cruciferous vegetable consumption and risk of cancers including lung [3], colorectal [4], and breast [5] cancers, all common cancers, mainly in developed and developing countries.

In animal-induced cancer model studies, PEITC has been reported to antagonise the carcinogenicity of chemicals in various tissues [6, 7]. It has exhibited a protection on chemically-induced carcinogenesis in the oesophagus, intestine, lung, and pancreas induced by azoxymethane, nitroso-compounds, and polycyclic aromatic hydrocarbons [6, 8–10], even though no beneficial effect was observed in colon cancer-induced by azoxymethane when the formation of aberrant crypt foci was used as biomarker [11]. Interestingly, the mercapturate of PEITC, an important metabolite, preserves its chemopreventive properties [12].

PEITC elicits its chemoprevention by blocking initiation [13] and post-initiation processes of tumour growth via modulating proliferation of cells cycle and induction of apoptosis [14–16]. Protecting against DNA damage and, thus, suppressing tumour initiation step is a major importance anticarcinogenic mechanism of isothiocyanates [17]. This can be achieved by reducing the availability of the metabolite products of chemical carcinogens by averting their generation, thus inhibition of their cytochrome P450-mediated bio-activation [18–20], and/or by stimulating their detoxification, via induction of enzyme systems such as the quinone reductase and glutathione *S*-transferases [21]. Nevertheless, the chemopreventive properties of isothiocyanates at this stage are multiple depending on dose regimen, animal species [22], nature of isothiocyanate [23], target tissue [24], and treatment protocol [25].

In studies employing precision-cut liver slices, the ability of PEITC to modulate carcinogen-metabolising enzymes in rat and human liver has been established [26, 27] and as a result revealed its potency to function as an anticancer agent. It is important to assess whether carcinogen metabolising enzymes react similarly to PEITC in animal models *in vivo* following exposure to low doses that simulate human dietary intake.

Most studies have focussed on quinone reductase and the glutathione *S*-transferases, and the modulation of other major carcinogen-metabolising hepatic enzyme systems by PEITC still remains to be evaluated. The objective of the current study was to evaluate in rats the effect of PEITC intake, employing dietary levels of exposure, on carcinogen metabolising enzymes systems, for example, epoxide hydrolase, glucuronosyl transferase, sulfotransferase, and N-acetyltransferase. Modulation of carcinogen metabolising enzymes was investigated in a liver tissue, as the liver is the principal site of the bioactivation of carcinogens [28]. A marked induction of epoxide hydrolase was observed at the high dose, while at the low and medium doses, glucuronosyl transferase and sulfotransferase levels were elevated, respectively.

## 2. Materials and Methods

Phenethyl isothiocyanate (PEITC) (LKT Laboratories, MN, USA), benzo[a]pyrene 4,5-epoxide and benzo[a]pyrene 4,5-diol (Mid-West Research Institute, KS, USA), 1-naphthol, 2-naphthol, and 4-aminobenzoic acid (Sigma Co. Ltd., Poole, Dorset, UK) were all purchased.

Male Wistar albino rats (180 ± 20 g) were obtained from B&K Universal Ltd (Hull, East Yorkshire, UK). The animals were housed at 22 ± 2°C, 30–40% relative humidity, in an alternating 12 h light: dark cycle with light onset at 07.00 h. After a week's acclimatization, the rats were randomly assigned into 4 groups of 5 rats each. Animal doses were chosen so that the low dose responses the average human daily intake of glucosinolates (75 mg/person/day or 1.07 mg/kg bw) [29]. Three groups were administered diets supplemented with 0.06 (low dose), 0.6 (medium dose), and 6.0 (high dose) *μ*mol PEITC/g diet, whereas one group served as control; animals were maintained on these diets for 14 days. At the end of the treatment period, rats were sacrificed and the livers were removed. Tissue samples were immediately frozen in liquid nitrogen and stored at −80°C until required. Hepatic S9 of liver (25% w/v), in 0.154 M KCl containing 50 mM Tris-HCl, pH 7.4, was prepared prior to microsomal and cytosolic separation by differential centrifugation. The following assays were carried out on isolated microsomes: glucuronosyl transferase (UDP) using 1-naphthol as substrate [30], epoxide hydrolase (EH) using benzo[a]pyrene 4,5-epoxide [31] as well as on the isolated cytosols, that is, sulfotransferase (SULT) using 2-naphthol as substrate [32], and N-acetyltransferase (NAT) using 4-aminobenzoic acid [33]; protein concentration was determined in both cellular subfractions using bovine serum albumin as standard [34].

Results are presented as mean ± standard deviation of groups of five rats each. Statistical evaluation was carried out by one-way ANOVA followed by the Dunnett's test.

## 3. Results and Discussion

The present study investigated the *in vivo* modulation of carcinogen-metabolising enzymes by PEITC, as this emerged as an impressing chemoprevention mechanism in the studies employing precision-cut rat [26] and human liver slices [27]. Rats were treated with diets supplemented with 3 different doses, 0.06 (low dose), 0.6 (medium dose), and 6.0 (high dose) *μ*mol PEITC/g diet for 14 days. The low dose is proportionate with human dietary intake of total glucosinolates, 75.5 mg/person/day or 1.07 mg/kg body weight for a 70 kg individual [29], which is comparable to an intake of 300 g watercress, the primary source of PEITC, based on 100 g watercress releasing approximately 25 mg PEITC [35]. Earlier studies in animal models have utilised either a single high dose or chronic intake of higher doses than those employed in the current study [36, 37]. Studies were carried out in the liver as it the principal site of bioactivation of chemical carcinogens [28].

**Figure 1:**
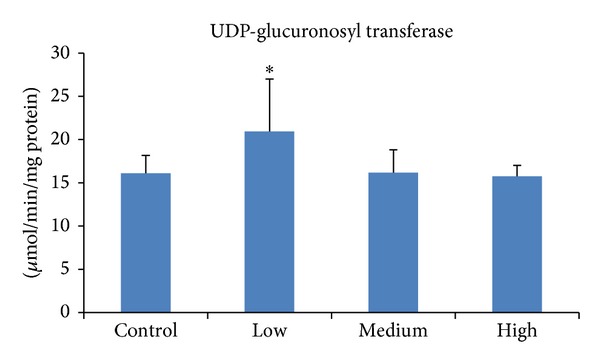
Effect of PEITC on glucuronosyl transferase activity in rat liver. Groups of five rats were exposed to diets supplemented with PEITC at 0.06 (low dose), 0.6 (medium dose), and 6.0 (high dose) *μ*mol/g diet for 14 days, whereas another group served as control. At the end of the treatment period, hepatic S9 was prepared from which microsomes were isolated and used to determine glucuronosyl transferase activity. Results are presented as mean ± SD for five rats. *P < 0.05.

Even though the effects of isothiocyanates on quinone reductase and glutathione *S*-transferases have been well reported both *in vitro* and *in vivo*, their capability to modulate other phase II enzyme systems involved in carcinogen metabolism has received little attention. Glucuronosyl transferases are a very essential phase II detoxifying enzyme system involved in the metabolism of chemical carcinogens including aromatic amines and polycyclic aromatic hydrocarbons [38]. The current studies found that PEITC at the low dose has the potential to upregulate glucuronosyl transferase (Figure 1), which commensurate previous findings where cruciferous vegetable consumption led to increased glucuronidation of the heterocyclic amine PhIP (2-amino-1-methyl-6-phenylimidazo [4,5-b]pyridine) in human volunteers, even though other components in the vegetables are likely to have also contributed to the upregulation of this enzyme [39]. Similarly, the consumption of diets supplemented with cruciferous vegetables reduced serum bilirubin levels, the glucuronidation that was catalysed by glucuronosyl transferase 1A1 (UGT1A1) [40]. In addition, PEITC isolated from watercress enhanced the metabolism of nicotine due to increased glucuronidation among smokers [41], while in rat liver slices, PEITC led to increase in the glucuronidation of 1-naphthol [26].

**Figure 2:**
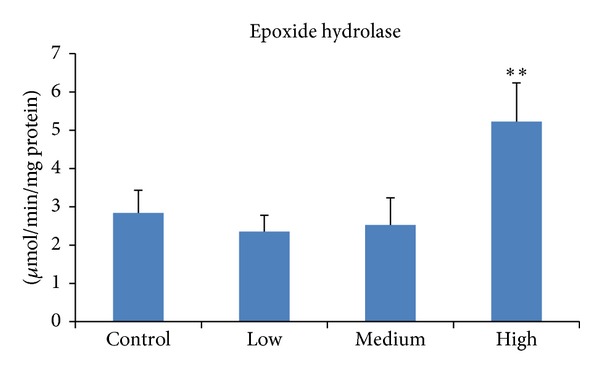
Effect of PEITC on epoxide hydrolase activity in rat liver. Groups of five rats were exposed to diets supplemented with PEITC at 0.06 (low dose), 0.6 (medium dose), and 6.0 (high dose) *μ*mol/g diet for 14 days, whereas another group served as control. At the end of the treatment period, hepatic S9 was prepared from which microsomes were isolated and used to determine epoxide hydrolase activity. Results are presented as mean ± SD for five rats. **P < 0.01.

Epoxide hydrolase is the phase II enzyme involved in the detoxification of many epoxides, the reactive intermediates of chemical carcinogens including polycyclic aromatic hydrocarbons, aflatoxin B1, and halogenated aliphatic compounds [42]. The present study showed that at the high dose PEITC elevated epoxide hydrolase (Figure 2), in concordance with our previous findings [26]; an 8-fold rise in the activity was exhibited, rendering it one of the potent inducers of this enzyme.

**Figure 3:**
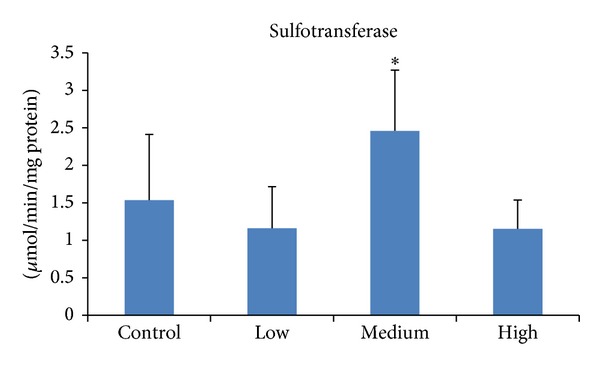
Effect of PEITC on sulfotransferase activity in rat liver. Groups of five rats were exposed to diets supplemented with PEITC at 0.06 (low dose), 0.6 (medium dose), and 6.0 (high dose) *μ*mol/g diet for 14 days, whereas another group served as control. At the end of the treatment period, hepatic S9 was prepared, and then cytosol was isolated and used to determine sulfotransferase activity. Results are presented as mean ± SD for five rats. *P < 0.05.

Sulfotransferases are the enzymes that catalyse sulfonation, an important reaction involved in the metabolism of numerous xenobiotics, drugs, and endogenous compounds [43]. The process of sulfonation encompasses the transfer of a sulfonyl (SO_3_^−^) group, normally to a hydroxyl on an acceptor molecule, which is catalysed by sulfotransferases [44]. It was revealed that medium dose of PEITC increased the level of this enzyme indicating that PEITC accepting sulfonyl group to its hydroxyl (Figure 3).

**Figure 4:**
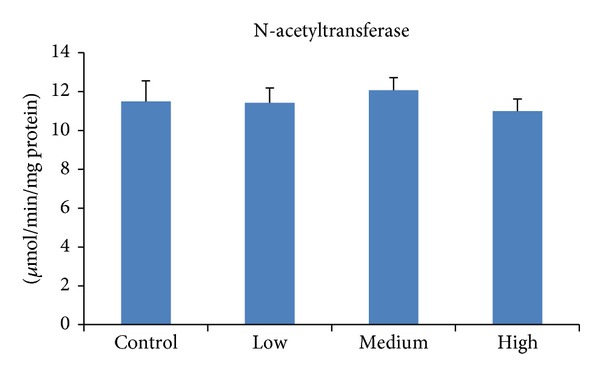
Effect of PEITC on N-acetyltransferase activity in rat liver. Groups of five rats were exposed to diets supplemented with PEITC at 0.06 (low dose), 0.6 (medium dose), and 6.0 (high dose) *μ*mol/g diet for 14 days, whereas another group served as control. At the end of the treatment period, hepatic S9 was prepared and then cytosol was isolated and used to determine N-acetyltransferase activity. Results are presented as mean ± SD for five rats.

N-acetyltransferases are cytosolic conjugating enzymes which transfer an acetyl group from acetyl coenzyme A to a xenobiotic acceptor substrate [45]. Our finding showed that this enzyme was unaffected (Figure 4), elucidating that acetyl group was transferred to PEITC forming N-acetylcysteine (NAC) conjugate of phenethyl isothiocyanate (PEITC-NAC), the major metabolite of PEITC that is abundant in watercress [46].

## 4. Conclusions

The present studies allow us to infer that phenethyl isothiocyanate modulates carcinogen-metabolising enzyme systems at doses reflecting human intake as a marked induction of epoxide hydrolase which was seen at the high dose, while at the low and medium doses, glucuronosyl transferase and sulfotransferase levels were upregulated, respectively. Increased levels of detoxification enzymes such as epoxide hydrolase, glucuronosyl transferase, and sulfotransferase are the likely one of the mechanisms for chemoprevention of PEITC.
